# Hierarchical PLGA/PEG Barrier Engineering of Alginate Hydrogels: Scale-Dependent Burst-Release Control in Beads and Microgels

**DOI:** 10.3390/biomimetics11050353

**Published:** 2026-05-20

**Authors:** Junseok Lee, Heeyoung Lee, Myeongjun Kim, Dae Gyu Song, Jaewon Jang, Jeong Koo Kim, Hong Jin Choi

**Affiliations:** 1 Institute of Nanobio Convergence, Pusan National University, Busan 46241, Republic of Korea; mydreamty1@gmail.com; 2 Department of Pharmacy, Inje University, Gimhae 50834, Republic of Korea; phylee1@inje.ac.kr; 3 Inje Institute of Pharmaceutical Sciences and Research, Inje University, Gimhae 50834, Republic of Korea; 4 Department of Biomedical Engineering, Inje University, Gimhae 50834, Republic of Korea; kaudwns00@gmail.com (M.K.); pio@piomedical.co.kr (D.G.S.); redwodnjs317@naver.com (J.J.)

**Keywords:** alginate hydrogel, PLGA/PEG coating, burst release, core–shell, dip-coating, scale-dependent release, hierarchical barrier, controlled drug delivery

## Abstract

Alginate hydrogels offer mild ionic gelation and tunable porosity for drug delivery, yet their hydrophilic, macroporous networks suffer from rapid initial burst release of water-soluble payloads. Here we introduce a hierarchical barrier-engineering strategy in which poly(D,L-lactide-co-glycolide)/poly(ethylene glycol) (PLGA/PEG) blend coatings are applied via dip-coating to Ca^2+^-cross-linked alginate beads (~1 mm) and microgels (~100 µm). For beads, three-cycle PLGA/PEG multilayer coating suppressed the initial swelling rate (dQ/dt) by ~50% and reduced 1 h burst release from >85% to ~60%, functioning as an “early-burst buffer” rather than a long-term depot. For microgels, a single PLGA/PEG layer partially attenuated burst release; however, an additional PLGA outer shell (double-barrier architecture) shifted the release-governing mechanism from swelling-dominated to diffusion-barrier-dominated control, limiting 10 min release to <10%. Core–shell formation was verified by confocal laser scanning microscopy (CLSM), scanning electron microscopy with energy-dispersive X-ray spectroscopy (SEM/EDS), Fourier-transform infrared spectroscopy (FT-IR), and X-ray photoelectron spectroscopy (XPS); thermogravimetric analysis (TGA) showed ~73–79% coating retention after 9 days in phosphate-buffered saline (PBS, 37 °C). A vacuum re-loading process further improved encapsulation efficiency (>50% for beads, >20% for microgels) without compromising gel integrity. In beads, burst control was governed by swelling suppression; in microgels, the additional PLGA shell shifted control to diffusion-barrier-dominated release, demonstrating that barrier architecture must be adapted to particle scale.

## 1. Introduction

Alginate hydrogels have been extensively studied as drug delivery matrices owing to their mild ionic gelation conditions, excellent biocompatibility, and tunable mechanical properties [1,2]. Alginate, an anionic polysaccharide derived from brown algae, forms a three-dimensional cross-linked network via ion exchange with multivalent cations such as Ca^2+^, following the well-known “egg-box” model. This process provides high water content, interconnected porous networks, and favorable drug-loading capacity [1,3,4]. Owing to these characteristics, alginate has found broad use in wound dressings, cell encapsulation, and localized drug delivery [2,5].

However, alginate hydrogels inherently suffer from rapid initial burst release of hydrophilic, low-molecular-weight drugs, attributable to their high water content and large pore architecture [4,6,7]. Water-soluble small-molecule drugs concentrated at or near the carrier surface are rapidly eluted within minutes upon immersion. This can cause local tissue toxicity, failure to maintain therapeutic drug levels, and poor reproducibility during formulation development [6,8,9]. Huang and Brazel [6] systematically reviewed that initial burst is determined by multiple factors including (1) high surface-area-to-volume ratio in small particles, (2) pore structure and boundary-layer diffusion, (3) insufficient drug–gel network interactions, and (4) concentration gradients and drying/rehydration history during fabrication.

Various strategies have been proposed to address the burst release problem. The most fundamental approaches involve modifying the matrix properties through alginate concentration and mannuronic-to-guluronic acid (M/G) ratio adjustment, or Ca^2+^ concentration optimization [1,4]. Complex gel formation with chitosan or poly-L-lysine, surface coating via layer-by-layer (LbL) deposition, and core–shell structure design have also been reported [10,11,12]. In particular, polymeric diffusion barriers introduced through coating enable release control that is difficult to achieve with a single matrix. Staged release patterns can be realized by independently designing the composition, thickness, and function of each layer [10,13].

Hierarchical multilayer structures arrange materials of differing permeability in series. This configuration offers a practical route to stepwise diffusion regulation by controlling both hydration pathways and diffusion rates [10,13,14]. Similar multilayer barrier principles operate in biological tissues: the stratum corneum–epidermis–dermis architecture of skin controls bidirectional molecular transport [15], and the intima–media–adventitia tri-layer of blood vessel walls governs selective permeation [16]. These natural examples have spurred interest in multilayer barrier design for drug delivery. Yet systematic application of hierarchical coating strategies to hydrogel carriers with direct comparison across particle scales has received little attention.

Poly(lactic-co-glycolic acid) (PLGA) is one of the most widely used biodegradable drug delivery polymers, supported by its FDA-approval history and extensive clinical experience [17,18]. PLGA degrades through hydrolysis of ester bonds, with degradation rates tunable from weeks to months depending on the lactide: glycolide molar ratio [17,19]. However, neat PLGA coatings excessively suppress moisture and ion permeation due to their high hydrophobicity. This can over-retard hydrogel core swelling and delay drug release until PLGA degradation onset, producing “long-term depot” behavior [18,20].

To overcome this limitation, blending poly(ethylene glycol) (PEG) with PLGA has been proposed [21,22]. PEG is widely known as a hydrophilic polymer with excellent water solubility and low protein adsorption [21,23]. Within PLGA matrices, PEG phase-separates to form hydrophilic channels (hydrated domains), thereby imparting moisture permeability [22,24]. Accordingly, PLGA/PEG blend coatings can realize a “semi-permeable barrier” that provides higher moisture and ion permeability than PLGA alone while maintaining hydrophobic diffusion barrier function. This makes them well suited for targeting short-term controlled release [22,24,25].

Particle scale is a critical variable governing drug release kinetics. Even for hydrogels of identical composition and structure, macroscale beads (~1 mm) and microscale microgels (~100 µm) differ by approximately 10-fold in surface-area-to-volume (SA/V) ratio. This leads to substantially different hydration rates, ion-exchange kinetics, and burst release magnitudes [4,26]. Because microgels hydrate and release drug much faster owing to their high SA/V ratio, a single coating strategy is unlikely to perform equally at both scales. However, systematic comparison of such scale-dependent release behavior in relation to barrier structure design remains insufficient [4,26,27]. Existing alginate coating approaches—chitosan complexation, polyelectrolyte LbL deposition, and single-polymer shell formation—have been applied at a fixed particle scale, and the relationship between barrier architecture and scale has not been addressed [10,11,12].

**Scheme 1:**
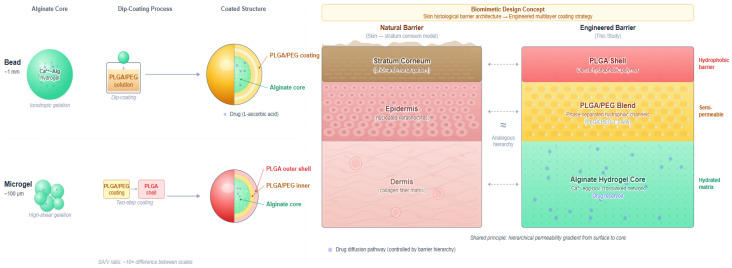
Hierarchical PLGA/PEG barrier engineering of alginate hydrogels. (**Left**) Fabrication of bead (~1 mm) and microgel (~100 µm) carriers. Ca^2+^-alginate cores were prepared by ionotropic gelation (beads) or high-shear gelation (microgels). Beads were coated by single-step PLGA/PEG dip-coating; microgels were coated in two steps to form a core–shell structure with a PLGA outer shell and a PLGA/PEG inner layer. Horizontal arrows indicate process flow; the SA/V ratio differs by approximately 10× between the two scales. L-ascorbic acid was used as the model drug. (**Right**) Biomimetic design concept comparing the skin barrier (stratum corneum/epidermis/dermis) with the engineered multilayer architecture. Color coding: pink, hydrophobic barrier (PLGA shell/stratum corneum); yellow, semi-permeable layer with phase-separated hydration channels (PLGA/PEG blend/epidermis); green, hydrated matrix (alginate core/dermis). The ≈ symbol denotes structural analogy between the natural and engineered systems; gray dots indicate the drug diffusion pathway.

**Scheme 2:**
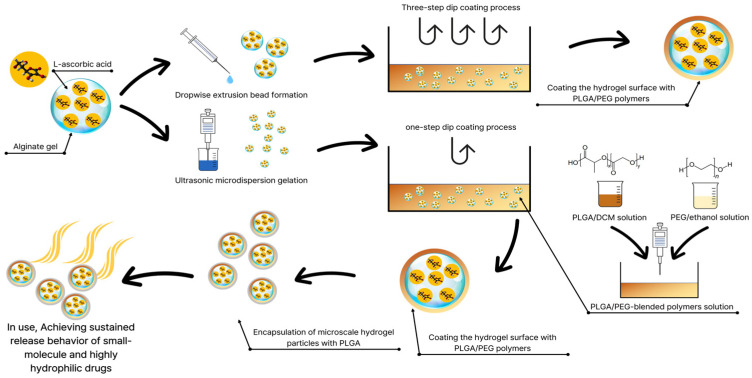
Alginate core fabrication (dropwise extrusion for beads, ultrasonic micro dispersion for microgels), PLGA/PEG blend dip-coating (3-step for beads, 1-step for microgels), PLGA shell formation for double-barrier microgels, and the resulting sustained-release concept.

Thus, the aim of the current study was to clarify how particle scale and barrier architecture jointly govern short-term release kinetics when PLGA/PEG blend-based hierarchical barriers are applied to alginate hydrogels (Scheme 1). An overview of the full experimental workflow, including fabrication, coating, and characterization, is presented in Scheme 2. To this end, Ca^2+^-ionically cross-linked alginate cores were used as a common platform, and PLGA/PEG barriers were applied at two distinct length scales: macroscale beads (~1 mm) and microscale microgels (~100 µm). Beads received PLGA/PEG multilayer dip-coating (3 cycles); microgels received either a single PLGA/PEG coating or an additional PLGA shell to form a double-barrier structure. Core–shell formation was verified by CLSM, SEM/EDS, FT-IR, and XPS, while coating stability was tracked by TGA over 1–9 days. Using L-ascorbic acid as a model drug, in vitro release testing, combined with mass-based swelling (dQ/dt) analysis, showed that the dominant release-control mechanism shifts from swelling-dominated to diffusion-barrier-dominated as particle scale decreases. We also applied a vacuum re-loading process and confirmed that it improves encapsulation efficiency (EE). Together, these findings provide a design framework for scale-customized, burst-buffered short-term controlled release from hydrogel carriers.

## 2. Materials and Methods

### 2.1. Materials

Sodium alginate (alginic acid sodium salt, medium viscosity, Sigma-Aldrich, St. Louis, MO, USA), calcium chloride dihydrate (CaCl_2_ 2H_2_O, ≥99%, Sigma-Aldrich), poly(D,L-lactide-co-glycolide) (PLGA, lactide:glycolide = 75:25, Mw ~10 kDa, Sigma-Aldrich), poly(ethylene glycol) (PEG, Mw ~8 kDa, Sigma-Aldrich), dichloromethane (DCM, ≥99.5%, Sigma-Aldrich), L-ascorbic acid (vitamin C, ≥99%, Sigma-Aldrich), FITC-dextran (70 kDa, Sigma-Aldrich), Nile Red (Sigma-Aldrich), poly(vinyl alcohol) (PVA, Mw ~31 kDa, 98–99%-hydrolyzed, Sigma-Aldrich), and phosphate-buffered saline (PBS, pH 7.4) were used. All reagents were used as received without further purification, and deionized water (18.2 MΩ cm) was used throughout the experiments.

### 2.2. Alginate Hydrogel Core Fabrication

#### 2.2.1. Macroscale Bead (~1 mm) Fabrication

Alginate aqueous solution (1–5% *w*/*v*; primary experimental group: 4% *w*/*v*) was dispensed dropwise into 100 mM CaCl_2_ solution using a syringe (21G needle) to induce external ionotropic gelation. Dispensed droplets were allowed to cure in the CaCl_2_ solution for 10–15 min, followed by two washes with PBS (+0.01% Tween-20) to remove residual Ca^2+^ and unreacted alginate. Bead diameters were measured using a digital caliper and confirmed to be approximately 1 mm (+/−0.1 mm, *n* = 30).

#### 2.2.2. Microscale Microgel (~100 µm) Fabrication

Alginate aqueous solution (2% *w*/*v*) was dispersed into 100 mM CaCl_2_ solution via high-shear external gelation, followed by fractionation through a micro-sieve (100 µm mesh) to ensure narrow size distribution. Laser diffraction particle size analysis yielded a median diameter (D50) of approximately 100 µm with a span value of 1.0–1.5, indicating a relatively uniform particle size distribution.

### 2.3. PLGA/PEG Blend Dip-Coating

PLGA (75:25, 10 kDa) and PEG (8 kDa) were dissolved in DCM:acetone (3:1 *v*/*v*)-mixed solvent to prepare blend coating solutions. The PLGA:PEG weight ratio was selected at 7:3 based on preliminary screening of macroscopic phase behavior across four compositions (6:4, 7:3, 8:2, and 9:1 *w*/*w*). Visual assessment revealed phase separation at the 6:4, 8:2, and 9:1 ratios, whereas only the 7:3 blend maintained acceptable phase compatibility with sufficient PEG content for hydrophilic channel formation (Figure S1). Total polymer concentration was fixed at 5 wt%.

For beads, Ca^2+^-alginate beads were immersed in the coating solution for 2 s, withdrawn, air-dried for 1 min at room temperature, and restabilized in 10 mM CaCl_2_ for 2 min; this sequence constituted one cycle, and three cycles were repeated to form multilayer coatings. For microgels, only one cycle was applied to prevent inter-particle bridging and aggregation.

### 2.4. Double-Barrier Microgel Fabrication (Additional PLGA Shell)

The double-barrier structure was fabricated using PLGA/PEG-coated microgels as precursors. These were dispersed in PLGA 5% (*w*/*v*) in DCM solution, and the PLGA shell was formed on the microgel exterior through solvent extraction/evaporation into 1% (*w*/*v*) PVA aqueous solution (~300 rpm, 30 min, room temperature). The final product was a tri-layer core–shell system comprising alginate core/PLGA-PEG inner layer/PLGA outer layer.

### 2.5. Coating Visualization and Chemical Verification

#### 2.5.1. CLSM (Confocal Laser Scanning Microscopy)

A dual-fluorescence labeling strategy was used to visualize the spatial distribution of the core–shell structure. The alginate core was labeled with FITC-dextran (70 kDa, 0.05% *w*/*v*), and the PLGA/PEG coating layer was labeled with Nile Red (1–2 mg/mL in DCM:acetone). Images were acquired on a Zeiss LSM800 (Carl Zeiss, Oberkochen, Germany) using sequential scan mode for FITC (ex/em: 488/515–540 nm) and Nile Red (ex/em: 561/600–650 nm) to prevent inter-channel crosstalk. Image processing and analysis were performed using ImageJ v1.54g (National Institutes of Health, Bethesda, MD, USA).

#### 2.5.2. SEM/EDS

Surface morphology and elemental composition of uncoated and PLGA/PEG-coated specimens were analyzed by field-emission scanning electron microscopy (FE-SEM; S-4800, Hitachi, Tokyo, Japan) equipped with energy-dispersive X-ray spectroscopy (EDS). Specimens were washed with deionized water, freeze-dried, mounted on aluminum stubs, and sputter-coated with platinum (~10 nm). SEM images were acquired at an accelerating voltage of 5 kV. Changes in carbon (C) and oxygen (O) atomic percentages and O/C ratios from EDS were used as indicators of coating layer formation.

#### 2.5.3. FT-IR

Fourier-transform infrared (FT-IR) spectra were acquired on a Nicolet iS50 spectrometer (Thermo Fisher Scientific, Waltham, MA, USA) equipped with an ATR accessory (diamond crystal). Spectra of uncoated and PLGA/PEG-coated specimens (freeze-dried) were recorded over the 4000–400 cm^−1^ range at 4 cm^−1^ resolution with 32 scans co-added. Analysis focused on (i) preservation of the alginate ionic cross-linked network (-COO- bands, ~1600–1650, and ~1400–1450 cm^−1^) and (ii) introduction of PLGA/PEG organic coating (PLGA C=O ~1750 cm^−1^, PEG C-O-C ~1100 cm^−1^). Peak deconvolution of the C 1s spectra was performed using CasaXPS v2.3.25 (Casa Software Ltd., Teignmouth, UK).

#### 2.5.4. XPS

X-ray photoelectron spectroscopy (XPS) was performed using a K-Alpha+ spectrometer (Thermo Fisher Scientific, Waltham, MA, USA) with a monochromatic Al K-alpha X-ray source (1486.6 eV). Survey spectra and high-resolution C 1s, O 1s, and Ca 2p spectra were acquired at pass energies of 200 eV and 50 eV, respectively. High-resolution C 1s spectra were deconvoluted to determine relative area ratios of C-C/C-H (284.8 eV) and C-O (286.3 eV) components. Changes in the C-C/C-H fraction and C 1s FWHM were used as indicators of PLGA/PEG coating introduction.

### 2.6. Thermogravimetric Analysis (TGA) for Coating Retention

PLGA/PEG-coated beads and microgels were stored in PBS (37 °C) and retrieved at days 1, 3, 5, 7, and 9 for thermogravimetric analysis (TGA; Q500, TA Instruments, New Castle, DE, USA). TGA was conducted under Ar atmosphere over 25–800 °C at a heating rate of 10 °C/min. Mass loss in the 200–400 °C range (∆W 200–400) was attributed to thermal decomposition of the PLGA/PEG organic coating, and the coating contribution was isolated by subtracting the 200–400 °C mass loss of uncoated controls under identical conditions. Coating retention (%) was calculated by normalizing each time point’s ∆W 200–400 value to the day 1 baseline (*n* = 5). Thermograms were analyzed using TA Universal Analysis v4.5A (TA Instruments, New Castle, DE, USA).

The 9-day monitoring period was chosen to verify coating stability well beyond the intended short-term release window (minutes to hours). Beyond 9 days, both beads and microgels underwent visible structural disintegration under physiological conditions (PBS, 37 °C), precluding further sampling (Figure S2).

### 2.7. Swelling Ratio and Initial Swelling Rate (dQ/dt) Measurement

Mass-based swelling ratio (Qt) was measured as a function of time for uncoated and PLGA/PEG-coated beads (~1 mm) and microgels (~100 µm). Specimens were immersed in PBS (pH 7.4, 37 °C, 100 rpm) and retrieved at predetermined time intervals (0, 5, 10, 20, 30, 60, 120, 180, 240, and 300 min); surface free water was removed by blotting, and mass (Wt) was measured immediately. Swelling ratio was defined as Qt (%) = (Wt − Wdry)/Wdry × 100. The initial swelling rate dQ/dt was calculated by finite difference between adjacent time points, and the first-interval rate (dQ/dt) (i.e., 0–5 min) was used to compare initial hydration suppression effects between uncoated and coated groups (*n* = 5). The 0–5 min interval was selected because it captures the steepest phase of hydration, during which the coating barrier effect is most pronounced and burst release is most rapid.

### 2.8. In Vitro Release Testing

L-ascorbic acid (vitamin C, Mw 176.12 Da) was selected as the model drug. As a highly water-soluble, low-molecular-weight hydrophilic molecule, L-ascorbic acid represents the “worst-case” burst release scenario in hydrogel matrices, exhibiting the fastest diffusion and maximum initial burst among candidate payloads [28,29]. Validating barrier efficacy under such extreme burst conditions provides the most stringent assessment of the coating strategy. If meaningful burst suppression is achieved, equivalent or superior performance can be anticipated for drugs of higher molecular weight or greater hydrophobicity.

#### 2.8.1. Beads: Uncoated vs. PLGA/PEG-Coated (2 Groups)

L-ascorbic acid (100 mM) was loaded into 4% (*w*/*v*) alginate beads via post-gelation soaking, and release tests were conducted in a two-group comparative design: uncoated vs. PLGA/PEG multilayer-coated (3 cycles). Release testing was performed in PBS (pH 7.4, 37 °C, 100 rpm) with complete medium replacement at predetermined intervals to maintain sink conditions. Sink volume was maintained at ≥50 mL per gram of gel; even at complete drug release, the resulting concentration would remain below 0.003% of L-ascorbic acid aqueous saturation solubility (~330 mg/mL at 20 °C), well within the conventional sink criterion. L-ascorbic acid concentration was quantified by UV-Vis spectrophotometry (V-550, JASCO Corporation, Tokyo, Japan) at 265 nm. Cumulative release (%) was calculated (*n* = 5).

#### 2.8.2. Microgels: Uncoated/PLGA/PEG-Coated/Double-Barrier (3 Groups)

Microgel-based release testing was designed as a three-group comparison: (i) uncoated microgels, (ii) PLGA/PEG single-barrier microgels, and (iii) PLGA/PEG + PLGA shell double-barrier microgels. L-ascorbic acid (100 mM) was loaded under identical conditions. Release conditions were the same as for beads: PBS (pH 7.4, 37 °C, 100 rpm), with UV-Vis quantification and cumulative release comparison (*n* = 5).

### 2.9. Model Drug Loading and Vacuum Re-Loading Process

Basic loading was performed by post-gelation soaking of prepared Ca^2+^-alginate beads and microgels in L-ascorbic acid (100 mM) aqueous solution for 2 h at room temperature. Vacuum re-loading was applied after basic loading: gels immersed in drug solution were subjected to an air-pressurized vacuum process (−85 to −90 kPa followed by return to 1 atm, 5 min/cycle, 3 cycles total) to remove residual gas from internal pores and induce convective drug penetration driven by the pressure differential. Encapsulation efficiency (EE) was defined as EE (%) = (M_actual_/M_theoretical_) × 100, and M_actual_ was determined from UV-Vis absorbance changes in the supernatant before and after loading (*n* = 5).

### 2.10. Statistical Analysis

All quantitative data were obtained from independent replicates (*n* = 5) and expressed as mean +/− standard deviation (SD). For two-group comparisons (beads: uncoated vs. coated), statistical significance was determined by Student’s *t*-test. For three-group comparisons (microgels: uncoated vs. single-barrier vs. double-barrier), one-way analysis of variance (ANOVA) followed by Tukey’s post hoc test was used. Significance was set at *p* < 0.05 for all analyses. All statistical analyses were performed using IBM SPSS Statistics v26.0 (IBM Corp., Armonk, NY, USA).

## 3. Results

### 3.1. Alginate Core Fabrication and Particle Size Characteristics

**Figure A1:**
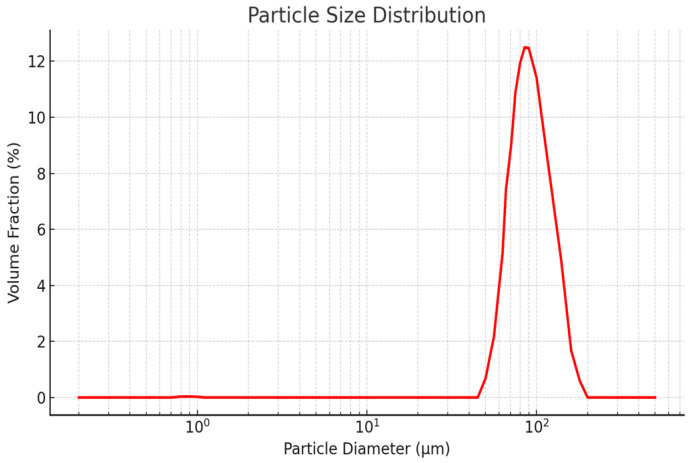
Particle size distribution (PSD) of alginate microgels measured by laser diffraction (Mastersizer 3000, Malvern Panalytical, Malvern, UK). The distribution exhibits a unimodal profile with D50 = 85.94 µm and a narrow span of 0.744, confirming uniform droplet formation during the emulsification process.

Macroscale beads fabricated by external ionotropic gelation exhibited a spherical morphology with a mean diameter of 1.0 +/− 0.1 mm as measured by digital caliper. Coated beads exhibited a slightly opaque appearance relative to uncoated counterparts, consistent with coating layer formation. Microscale microgels showed a median diameter (D50) of approximately 100 µm with a span of ~1.2 by laser diffraction analysis, indicating a relatively uniform distribution (Figure A1, Table A1). Structural stability (disintegration time) was evaluated across 1–5% (*w*/*v*) alginate concentrations under PBS (37 °C) immersion. Both beads and microgels showed concentration-dependent increases in stability, and PLGA/PEG coating further improved structural retention, particularly at lower concentrations (1–2% *w*/*v*).

### 3.2. Core–Shell Structure Visualization by CLSM

**Figure 1:**
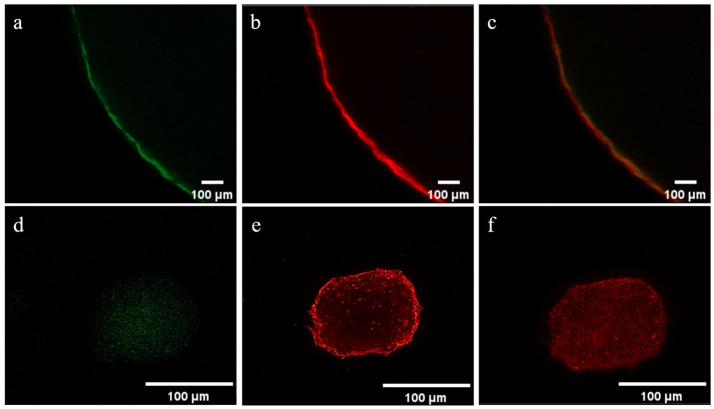
CLSM core–shell visualization of PLGA/PEG-coated alginate hydrogels. Bead: (**a**) FITC-dextran (alginate core, green), (**b**) Nile Red (PLGA/PEG coating, red), (**c**) merged. Microgel: (**d**) FITC-dextran, (**e**) Nile Red, (**f**) merged. Scale bars = 100 µm. Representative images from *n* = 3 independent specimens per condition.

Dual-fluorescence CLSM images using FITC-dextran (green) and Nile Red (red) revealed clearly separated core–shell morphologies in both beads and microgels, with the alginate core (green) and PLGA/PEG coating layer (red) distinctly resolved. For beads, FITC-dextran (Figure 1a) and Nile Red (Figure 1b) channels showed uniform coating layers resulting from three dip-coating cycles, confirmed in the merged image (Figure 1c). Microgels also showed localized FITC (Figure 1d) and Nile Red (Figure 1e) signals confined to the core and particle periphery, respectively, supporting surface-limited coating formation (Figure 1f). In double-barrier microgels, a dual fluorescent ring pattern was visible. The PLGA/PEG inner layer and the PLGA shell outer layer were resolved as separate rings, confirming hierarchical multilayer structure formation.

### 3.3. Surface Morphology and Elemental Composition by SEM/EDS

**Figure 2:**
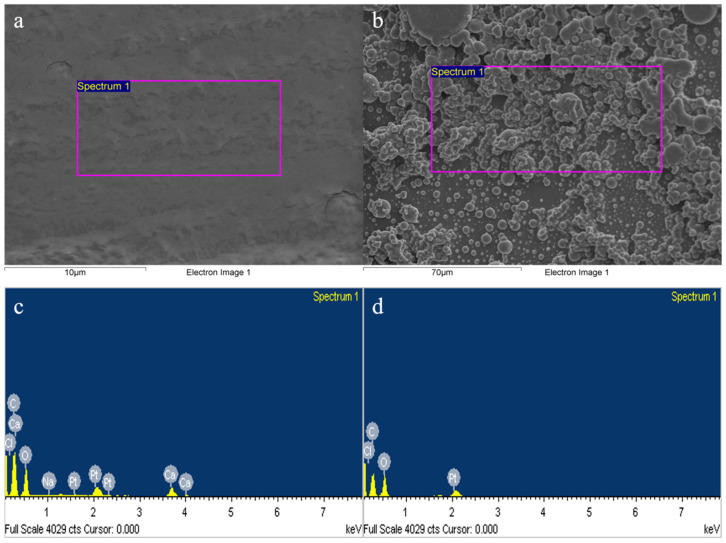
SEM/EDS surface morphology and elemental analysis. SEM micrographs of (**a**) uncoated and (**b**) PLGA/PEG-coated alginate hydrogel surfaces. EDS spectra of (**c**) uncoated and (**d**) coated specimens from the regions indicated by pink rectangles in (**a**,**b**). Representative images from *n* = 3 independent specimens per condition.

SEM observation revealed that uncoated alginate hydrogels exhibited relatively smooth surfaces, whereas PLGA/PEG-coated hydrogels displayed granular, multilayer-like surface textures (Figure 2a,b). EDS spectra (Figure 2c,d) showed relative increases in C atomic % and corresponding changes in O/C ratio for coated specimens (Table 1). These shifts support surface introduction of the organic coating layer.

### 3.4. FT-IR Analysis of Alginate-PLGA-PEG Characteristic Bands

**Figure 3:**
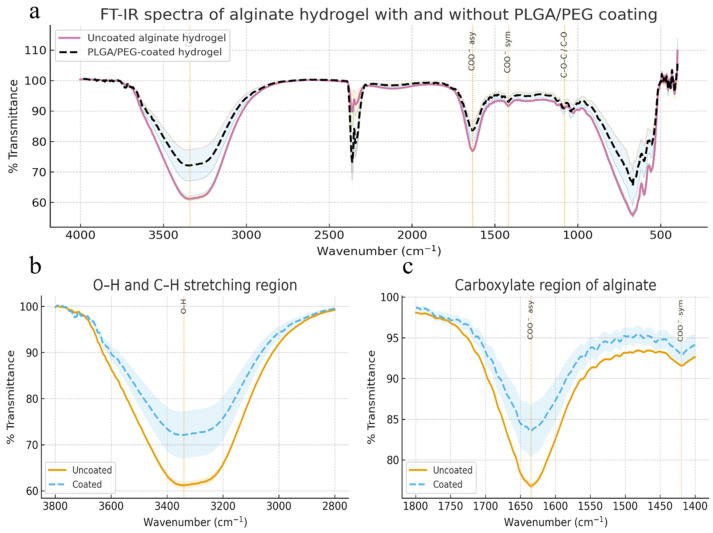
FT-IR spectra of uncoated and PLGA/PEG-coated alginate hydrogels. (**a**) Full-range spectra (4000–400 cm^−1^); (**b**) expanded O-H and C-H stretching region (3800–2800 cm^−1^); (**c**) carboxylate region of alginate (1800–1400 cm^−1^). In all panels, solid lines represent uncoated samples and dashed lines represent PLGA/PEG-coated samples (color coding shown in each panel legend); shaded bands around each curve indicate the standard deviation (*n* = 3). Vertical dashed lines indicate the positions of key characteristic bands as labeled in each panel (COO^−^ asymmetric and symmetric stretching, C-O-C stretching, and O-H stretching).

Full-range FT-IR spectra (Figure 3a) of coated specimens showed the appearance or relative intensity increase in the PLGA carbonyl band (C=O, ~1750 cm^−1^) and PEG ether band (C-O-C, ~1100 cm^−1^). These changes verify surface introduction of PLGA/PEG components. Meanwhile, alginate carboxylate (-COO-) bands (asymmetric ~1600–1650 cm^−1^, symmetric ~1400–1450 cm^−1^) showed negligible changes in peak position and shape after coating, confirming preservation of the Ca^2+^-alginate ionic cross-linked network through the coating process. The expanded O-H/C-H stretching region (Figure 3b) revealed relative attenuation of the broad O-H band and increased C-H stretching (2800–3000 cm^−1^) intensity in coated specimens. The carboxylate region (Figure 3c) confirmed preserved COO^−^ peak positions, consistent with changes in surface hydration environment due to organic layer coverage.

### 3.5. Surface Chemical Composition by XPS

**Figure 4:**
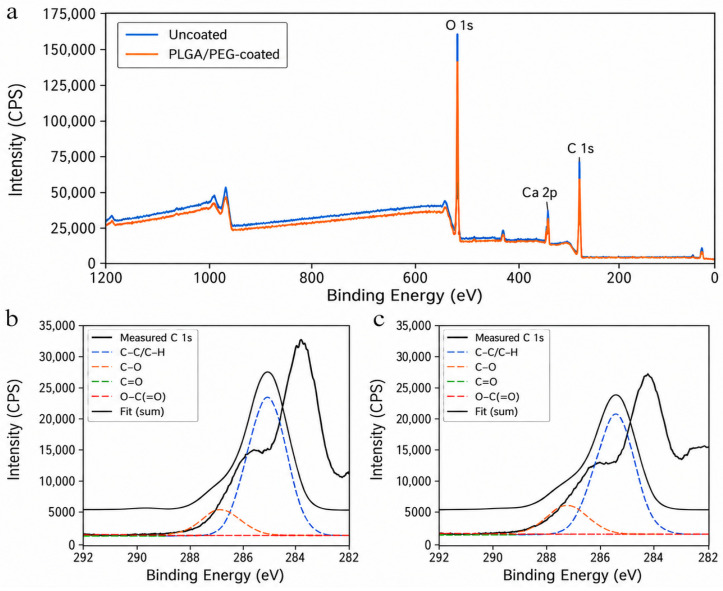
XPS analysis of uncoated and PLGA/PEG-coated alginate hydrogels. (**a**) Survey spectra showing O 1s, C 1s, and Ca 2p peaks; the coated specimen exhibits increased C 1s intensity and attenuated Ca 2p intensity relative to the uncoated control. (**b**) C 1s high-resolution spectrum of uncoated specimen, deconvoluted into C-C/C-H (284.8 eV) and C-O (286.3 eV) components. (**c**) C 1s high-resolution spectrum of PLGA/PEG-coated specimen, showing an increased C-C/C-H fraction (69.8% → 75.5%) and broadened FWHM (1.56 → 2.63 eV) relative to the uncoated control. Representative spectra from *n* = 5 independent specimens per condition.

XPS survey spectra of coated specimens showed increased C 1s intensity and attenuated Ca 2p intensity relative to uncoated controls, consistent with organic coating layer formation at the surface (Figure 4a). In high-resolution C 1s spectra (Figure 4b,c), uncoated specimens showed C-C/C-H (284.8 eV) and C-O (286.3 eV) components characteristic of the alginate saccharide backbone. After coating, the C-C/C-H fraction increased from 69.8% to 75.5% of the total C 1s area, and the C 1s FWHM broadened from 1.56 to 2.63 eV (+68%), both attributable to aliphatic and ester carbon introduced by the PLGA/PEG overlayer. The contrasting elemental shifts between EDS and XPS reflect their different sampling depths. EDS probes ~1–2 µm into the sample, well beyond the thin coating, so the alginate substrate dominates and C atomic % barely changes (+0.19 percentage points: 50.79% → 50.98%). XPS samples only the outermost ~10 nm and detects a ~10-fold larger shift (+2.03 percentage points: 55.96% → 57.99%). Because both PLGA/PEG and alginate are composed primarily of C and O, C atomic % alone is not a sensitive coating indicator. The C/O ratio increase (1.367 → 1.477, +8.0%) and Ca 2p decrease (3.11% → 2.75%, −11.6%) provide more diagnostic evidence of coating formation. Ca originates exclusively from Ca^2+^-crosslinked alginate and is absent in PLGA/PEG; its attenuation therefore directly reflects overlayer shielding of the substrate. Excluding Cyieldrenormalizing the organic components yields an apparent surface composition of C 59.6% and O 40.4% for the coated specimen, compared with C 57.8% and O 42.2% for the uncoated control. The coated values approach the theoretical PLGA/PEG (7:3 *w*/*w*) blend composition (C ~60.5%, O ~39.5%), indicating that the surface within the XPS sampling depth is partially occupied by the coating material.

### 3.6. TGA-Based Coating Retention Assessment

**Figure 5:**
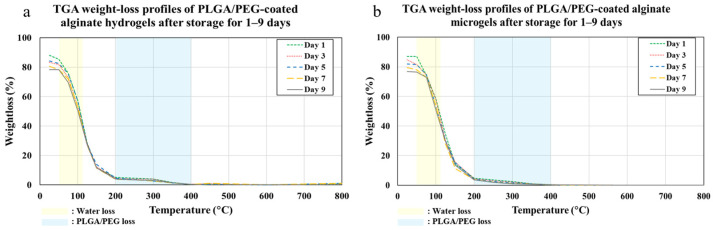
TGA weight-loss profiles of PLGA/PEG-coated alginate hydrogels after 1–9 days of storage in PBS at 37 °C. (**a**) Beads, (**b**) microgels. Yellow shading = water loss region; blue shading = PLGA/PEG decomposition region (200–400 °C). Representative curves from *n* = 5 per time point.

Analysis of TGA curves (∆W 200–400 °C) for PLGA/PEG-coated beads and microgels over 1–9 days of PBS (37 °C) immersion revealed gradual coating decrease with increasing storage time, yet without abrupt structural collapse (Figure 5a,b; Table 2). At day 9, coating retention was approximately 73% for beads (Figure 5a) and 79% for microgels (Figure 5b). Of note, microgels retained comparable or slightly higher coating levels than beads despite their larger SA/V ratio. Coating loss therefore appears to depend not only on specific surface area but also on coating continuity, surface bonding, and selective erosion at defect-prone sites during moisture and ion exposure. The smaller and more regular surface geometry of microgels may promote more uniform coating deposition with fewer defect sites, contributing to slightly higher retention despite the larger SA/V ratio.

TGA-based coating retention quantifies the remaining organic mass attributable to PLGA/PEG but does not distinguish between a uniformly intact barrier and one that has partially eroded with mass redistribution. The retention values therefore indicate that most coating material persists, not that barrier function is uncompromised. All stability tests were conducted under static PBS immersion at 37 °C; dynamic environments (mechanical agitation, peristalsis, blood flow) could accelerate coating loss.

### 3.7. Swelling Behavior: Beads and Microgels

#### 3.7.1. Beads

**Figure 6:**
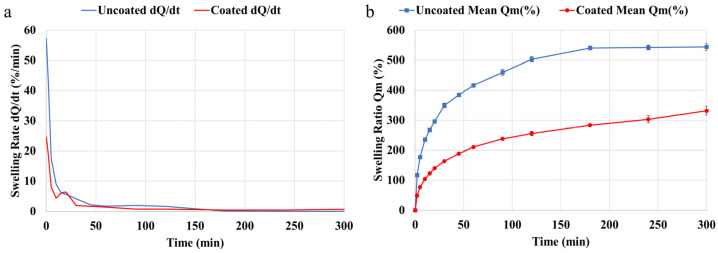
Swelling behavior of uncoated and PLGA/PEG-coated alginate beads in PBS (37 °C). (**a**) Initial swelling rate (dQ/dt) as a function of time; the coated group shows a reduced peak rate in the 0–5 min interval compared to the uncoated group. (**b**) Mean swelling ratio (Qm) over 300 min; error bars represent standard deviation (*n* = 5).

Uncoated beads showed rapid water uptake immediately upon immersion with an initial dQ/dt of approximately 55% min^−1^, whereas PLGA/PEG-coated beads showed a reduced rate of approximately 25% min^−1^, indicating suppression of initial hydration rate by approximately half (Figure 6a). Both groups converged to dQ/dt < 1% min^−1^ after 180 min. Swelling ratio also differed between groups (Figure 6b). Uncoated beads reached approximately 235% at 10 min and 560% at 300 min, whereas coated beads reached only approximately 100% and 320% at the same time points. Long-term equilibrium swelling ratio was also maintained lower for coated beads (~430–440%) compared to uncoated.

#### 3.7.2. Microgels

**Figure 7:**
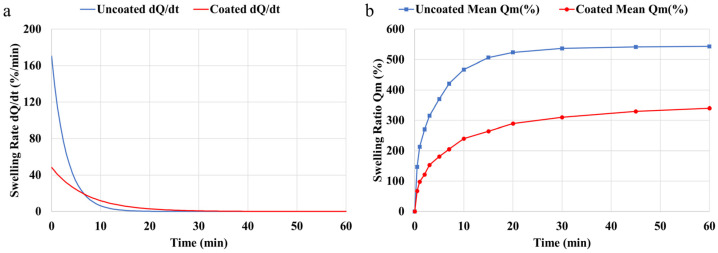
Swelling behavior of uncoated and PLGA/PEG-coated alginate microgels in PBS (37 °C). (**a**) Initial swelling rate (dQ/dt) as a function of time; both groups reach near-zero rates within ~30 min, but the coated group shows a lower initial peak. (**b**) Mean swelling ratio (Qm) over 60 min; error bars represent standard deviation (*n* = 5).

Microgels (~100 µm) exhibited inherently faster hydration due to their high SA/V ratio. Uncoated microgels showed initial dQ/dt values up to approximately 200% min^−1^, swelling beyond 500% within 10 min and approaching equilibrium within 30 min (Figure 7a). PLGA/PEG-coated microgels showed initial dQ/dt reduction to approximately 100% min^−1^, confirming initial hydration suppression, with 10 min swelling ratio reduced to approximately 250% (Figure 7b). Coated microgel swelling continued more gradually, reaching an equilibrium value of approximately 350% around 150 min.

### 3.8. In Vitro Release Behavior

#### 3.8.1. Beads: Uncoated vs. PLGA/PEG-Coated

**Figure 8:**
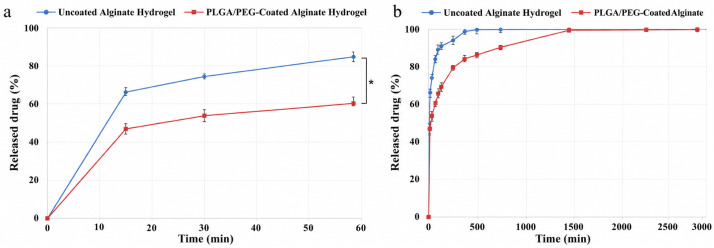
In vitro L-ascorbic acid release from uncoated and PLGA/PEG-coated alginate beads. (**a**) Initial 60 min profile (* *p* < 0.05), (**b**) full 24 h (3000 min) profile. Error bars = SD (*n* = 5).

Cumulative L-ascorbic acid release from uncoated 4% (*w*/*v*) alginate beads exhibited typical burst patterns: 65–70% at 15 min, ~75% at 30 min, and >85% at 1 h (Figure 8a). In contrast, PLGA/PEG-coated beads showed approximately 45%, 50%, and 60% at the same time points, representing a 20–25 percentage-point reduction in 1 h burst release (Student’s *t*-test at 1 h cumulative release, * *p* < 0.05), confirming the coating’s burst attenuation effect. In the 1–8 h interval, the release curve slope for the coated group became more gradual with sustained release, and by 24 h, release converged to near-complete levels regardless of coating presence (Figure 8b). The PLGA/PEG coating thus functions not as a long-term release depot but as an “early-burst buffer.” It temporarily modulates surface water influx and diffusion pathways during the initial period of rapid hydration and swelling.

#### 3.8.2. Microgels: Uncoated vs. Single Barrier vs. Double Barrier

**Figure 9:**
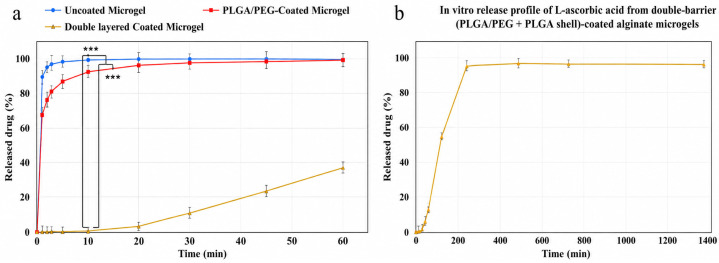
In vitro L-ascorbic acid release from alginate microgels. (**a**) Initial 60 min comparison of uncoated, PLGA/PEG single-barrier, and double-barrier microgels (*** *p* < 0.001), (**b**) full 24 h profile of double-barrier microgels. Error bars = SD (*n* = 5).

In microgels, the release control effect of barrier architecture was even more pronounced (Figure 9a). Uncoated microgels exhibited rapid burst release (~80% within 10 min), while PLGA/PEG single-coated microgels showed partial suppression (~55% at 10 min). The most notable result was that PLGA/PEG + PLGA shell double-barrier microgels limited 10 min release to <10% and 60 min release to approximately 30–40%, requiring several hours to reach 90–95% cumulative release (one-way ANOVA/Tukey at 10 min cumulative release, *** *p* < 0.001 vs. uncoated and single-barrier groups) (Figure 9b). The data confirm that single PLGA/PEG coating alone is insufficient for adequate burst suppression in high-SA/V microgels. Placement of an additional hydrophobic PLGA shell as the outermost layer creates a “hydration induction period” that shifts the release-governing mechanism to diffusion-barrier-dominated control.

### 3.9. Encapsulation Efficiency Enhancement by Vacuum Re-Loading

**Figure 10:**
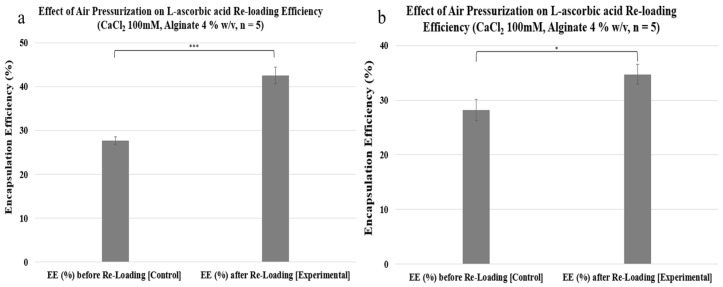
Effect of vacuum re-loading on L-ascorbic acid encapsulation efficiency (EE). (**a**) Beads (*** *p* < 0.001), (**b**) microgels (* *p* < 0.05). CaCl_2_ 100 mM, alginate 4% *w*/*v*, *n* = 5.

Comparison of vacuum re-loading with basic soaking showed significant EE improvements. For beads, EE increased from approximately 32% (basic soaking) to approximately 51% (vacuum re-loading), corresponding to a >50% relative improvement (Figure 10a). For microgels, EE increased from approximately 18% to approximately 23%, a >20% relative improvement (Figure 10b). Gel morphology and mechanical stability were maintained after vacuum re-loading, indicating that convective penetration driven by the pressure of differential improved drug access to internal pores without harming gel integrity.

## 4. Discussion

### 4.1. Redefining the Role of PLGA/PEG Coating: “Early-Burst Buffer”

The release data indicate that the PLGA (75:25)/PEG (8 kDa) blend coating does not act as a month-scale long-term depot. Instead, it serves as an “early-burst buffer” that slows the initially rapid hydration, swelling, and diffusion over several hours. Three observations support this interpretation. First, coated and uncoated beads converge to near-complete release within 24 h. Second, the coating behaves as a semi-permeable rather than impermeable barrier—it retards but does not block hydration and ion exchange. Third, TGA retention remains at ~70–80% after 9 days, confirming that the coating stays physically intact over the short term.

This behavior reflects the PLGA/PEG blend ratio (7:3 *w*/*w*): PEG domains create hydration channels that raise coating permeability well above that of neat PLGA [22,24]. The PLGA continuous phase still provides hydrophobic diffusion resistance, but the PEG dispersed phase lets moisture and ions partially penetrate. The overall coating therefore acts as a ‘gradual attenuation’ barrier rather than an ‘on/off’ blockade, suited to short-term controlled release on the hours-to-one-day timescale [24,25]. In vivo pharmacokinetic parameters were not assessed in the present study. Nevertheless, this hour-scale attenuation of the initial burst may be relevant for drugs with a narrow therapeutic index, where moderating the early release peak could help limit dose-dumping-associated toxicity.

For context, representative data from the literature show a range of burst-release outcomes with alginate-based coating strategies: a chitosan/alginate hydrogel released ~35% of hydrocortisone and ~65% of sulfasalazine within 1 h, with the difference attributable to drug hydrophilicity [30]; five-layer LbL-coated alginate beads sustained ATP release over 14 days [31]; and nano-coated alginate hydrogels eliminated VEGF burst entirely [32]. Our bead results (~60% at 1 h) fall in the mid-range for single-strategy coatings applied to hydrophilic small molecules, while the double-barrier microgel results (<10% at 10 min) are competitive with multilayer approaches.

### 4.2. Scale-Dependent Release Mechanism Transition

The same PLGA/PEG coating strategy engaged different release control mechanisms depending on particle scale.

In mm-scale beads, PLGA/PEG multilayer coating alone simultaneously reduced initial swelling rate and burst release by approximately 50%. This parallel suppression is consistent with a “swelling-dominated burst control” mechanism: at the relatively low SA/V ratio of beads, hydration delay by the coating directly translates to release suppression. The coating retards water and ion influx, slowing network swelling and ion exchange, thereby producing a cascade of reduced pore expansion and drug diffusion.

In contrast, for µm-scale microgels, PLGA/PEG single coating reduced the initial swelling rate by a similar proportion, yet burst release did not decrease proportionally. The high SA/V ratio of microgels (~10× that of beads) apparently overwhelms swelling suppression alone, allowing rapid diffusion across the large total surface. When a PLGA shell was added to form a double barrier, however, burst dropped sharply—evidence that the dominant release-control factor shifted from “swelling rate” to “diffusion resistance.” This shift is consistent with a series diffusion-resistance models: in core–shell structures, the effective mass transfer coefficient (k_eff_) scales as 1/k_eff_ ~ sum(δ_i_/D_i_), where δ_i_ and D_i_ are each layer’s thickness and diffusion coefficient, respectively [13]. Even a thin PLGA shell with a low diffusion coefficient can therefore raise total diffusion resistance considerably.

Microgels were prepared at 2% (*w*/*v*) alginate, producing a less dense network than the 4% (*w*/*v*) beads. Despite this lower matrix density, double-barrier microgels achieved the strongest burst suppression among all groups, indicating that barrier layer resistance, not matrix density, is the dominant factor at this scale.

We note that this proposed mechanism transition from swelling-dominated to diffusion-barrier-dominated control is inferred from the convergence of swelling and released data across two particle scales, rather than directly measured through independent decoupling experiments. Definitive verification using non-swelling matrices as controls remains an important direction for future work.

### 4.3. Structural Analogy with Natural Multilayer Barrier Systems

The hierarchical barrier structure implemented in this study shares structural and functional similarity with multilayer diffusion-regulation systems observed in nature. In biological tissues, molecular transport is often regulated in a stepwise manner by serial arrangement of multiple layers with different permeabilities rather than by a single homogeneous membrane [15,16]. For example, the stratum corneum of skin, composed of highly hydrophobic ceramide-lipid matrix, primarily blocks external substance penetration. The underlying viable epidermis and dermis secondarily regulate diffusion within a hydrophilic matrix [15]. This alternating arrangement of hydrophobic and hydrophilic layers enables selective, stepwise transport control that is difficult to achieve with a single homogeneous barrier.

In our double-barrier microgels, the tri-layer structure of (i) alginate core (high permeability, hydrophilic), (ii) PLGA/PEG inner layer (intermediate permeability, semi-permeable), and (iii) PLGA shell outer layer (low permeability, hydrophobic) mirrors this natural “graded-permeability” principle. The outermost hydrophobic PLGA shell creates a “hydration induction period” that delays core hydration, while the inner PLGA/PEG semi-permeable membrane continues to modulate drug diffusion even after water reaches it. This dual mechanism functionally parallels the hierarchical role division where the stratum corneum delays initial moisture penetration and the underlying tissue regulates subsequent diffusion. This comparison is intended as a structural/functional analogy, not an experimentally quantified equivalence. Measuring independent permeation coefficients for each synthetic layer and comparing them with published biological barrier data remains to be done.

### 4.4. Practical Significance of Vacuum Re-Loading

While post-gelation soaking is straightforward, EE is limited by residual bubbles and pore accessibility constraints within the gel. The vacuum re-loading process introduced in this study operates by (1) removing residual gas from the gel interior during depressurization and (2) inducing convective drug penetration driven by the pressure differential during repressurization [33]. The resulting EE values were ~51% for beads and ~23% for microgels, corresponding to relative gains of >50% and >20%, respectively. Gel morphology and mechanical stability were maintained after the process. For context, post-gelation soaking of alginate beads with hydrophilic small molecules typically yields EE in the 20–50% range [4,6]; the EE values obtained here are therefore within the expected range for this loading method. The PLGA/PEG coating is applied after drug loading and does not restrict EE; its role is to modulate release kinetics, not loading capacity.

### 4.5. Limitations and Future Directions

This study is limited to a single hydrophilic, low-molecular-weight model drug, L-ascorbic acid (Mw 176 Da). However, L-ascorbic acid represents the worst-case burst release scenario in hydrogel matrices [28,29]; thus, the barrier efficacy observed here can be viewed as a conservative estimate. Whether this barrier strategy extends to drugs of higher molecular weight (1–50 kDa) or different polarities remains to be tested. Charged biomolecules may interact electrostatically with the anionic alginate network, adding complexity not addressed in this study.

Coating thickness and surface roughness were not directly quantified in this study. Alginate hydrogels contain >90% water, and the shrinkage caused by dehydration during sample preparation precludes reliable cross-sectional dimensional measurements. Instead, coating retention was tracked over 9 days by TGA (Table 2). Cryo-SEM/FIB or environmentally controlled AFM in the hydrated state would be required to obtain accurate thickness and roughness data, and this remains a priority for future work. Coating microstructure after vacuum cycling was not examined in this study; whether repeated pressure cycling affects coating continuity at the sub-surface level warrants investigation in future work.

In vivo release testing and cytotoxicity evaluation were beyond the scope of this study, which focused on establishing fundamental structure–property–release relationships. Given the established biocompatibility profiles of PLGA [17,18], PEG [21], and alginate [1,2], the next steps would include cytotoxicity testing (ISO 10993-5-based MTT/CCK-8 assay [34]), tissue compatibility evaluation, and in vivo pharmacokinetic studies [35]. Based on the particle sizes and release characteristics, plausible application spaces include injectable local-delivery depots (microgels), oral systems requiring burst control during gastric transit (beads), and implantable hydrogel matrices for wound or surgical sites. Independent measurement of each layer’s permeation coefficient and fitting to multi-compartment diffusion models would enable quantitative mechanistic verification [36,37]. Stimuli-responsive coatings that offer selective, adaptive permeation control are also worth exploring [38].

## 5. Conclusions

We applied PLGA/PEG blend-based hierarchical barriers to Ca^2+^-cross-linked alginate beads (~1 mm) and microgels (~100 µm) and asked how particle scale and barrier architecture govern short-term release kinetics.

Complementary analyses by CLSM, SEM/EDS, FT-IR, and XPS confirmed core–shell structure formation and Ca^2+^-alginate network preservation in both beads and microgels upon PLGA/PEG coating. TGA showed coating retention of approximately 73% (beads) and 79% (microgels) after 9 days in PBS at 37 °C, sufficient for short-term applications.

The PLGA/PEG coating acted not as a complete long-term release depot but as an “early-burst buffer,” reducing 1 h burst from >85% to ~60% in beads while allowing near-complete release within 24 h.

The release data revealed a scale-dependent transition in the dominant control mechanism. In mm-scale beads, the coating suppressed swelling and burst in parallel, indicating swelling-dominated control. In µm-scale microgels, swelling suppression alone could not overcome the high SA/V ratio; only the double-barrier structure with an additional PLGA shell shifted control to diffusion-barrier-dominated release, limiting 10 min release to <10% and 60 min release to ~30–40%.

Vacuum re-loading raised EE from ~32% to ~51% for beads and from ~18% to ~23% for microgels without damaging gel structure.

Hierarchical PLGA/PEG barrier engineering, when tailored to particle scale, can thus serve as a workable route to burst-buffered short-term controlled release on the minutes-to-hours timescale.

## Figures and Tables

**Table 1:** EDS elemental composition of alginate hydrogels before and after PLGA/PEG coating.

	Uncoated Hydrogel		PLGA/PEG-Coated Hydrogel	
	C	O	C	O
Weight (%)	32.46	34.83	30.98	37.06
Atomic (%)	50.79	40.92	50.98	45.78

Values represent atomic and weight percentages obtained from EDS area analysis of the regions indicated in Figure 2a,b.

**Table 2:** TGA-derived ΔW_200–400_ °C values (wt%) and coating retention of PLGA/PEG-coated alginate hydrogels during storage (day 1–9, PBS, 37°C, *n* = 5).

Sample	Day 1	Day 3	Day 5	Day 7	Day 9	Retention (%)
Beads	4.630	4.205	4.164	3.467	3.393	~73
Microgels	4.313	3.954	3.662	3.566	3.397	~79

Retention (%) was calculated by normalizing each time point’s ΔW_200–400_ value to the day 1 baseline.

## Data Availability

The data presented in this study are available on request from the corresponding authors.

## Supplementary Materials

The following supporting information can be downloaded at https://www.mdpi.com/article/10.3390/biomimetics11050353/s1, Figure S1: Visual comparison of macroscopic phase behavior in PLGA:PEG blend solutions at different mixing ratios (6:4, 7:3, 8:2, and 9:1 *w*/*w*) in DCM:acetone (3:1 *v*/*v*) mixed solvent at a total polymer concentration of 5 wt%; Figure S2: Photographs of PLGA/PEG-coated alginate beads and microgels after 1, 3, 5, 7, and 9 days of storage in PBS at 37 °C, showing progressive swelling and structural degradation.

## Abbreviations

The following abbreviations are used in this manuscript:

AFM	atomic force microscopy
ANOVA	analysis of variance
ATR	attenuated total reflectance
CCK-8	Cell Counting Kit-8
CLSM	confocal laser scanning microscopy
DCM	dichloromethane
EDS	energy dispersive X-ray spectroscopy
EE	encapsulation efficiency
FIB	focused ion beam
FITC	fluorescein isothiocyanate
FT-IR	Fourier-transform infrared spectroscopy
MTT	3-(4,5-dimethylthiazol-2-yl)-2,5-diphenyltetrazolium bromide
PBS	phosphate-buffered saline
PEG	poly(ethylene glycol)
PLGA	poly(lactic-co-glycolic acid)
PVA	poly(vinyl alcohol)
SA	sodium alginate
SD	standard deviation
SEM	scanning electron microscopy
TEM	transmission electron microscopy
TGA	thermogravimetric analysis
UV	ultraviolet
XPS	X-ray photoelectron spectroscopy

## Appendix A

**Table A1:** Summary of particle size distribution (PSD) parameters for alginate microgels measured by laser diffraction.

**Parameter**	**Value**
D10 (µm)	61.92
D50 (µm)	85.94
D90 (µm)	125.88
D [4, 3] (µm)	89.86
D [3, 2] (µm)	74.36
Mode (µm)	85
Span	0.744
Fraction 80–120 µm (%)	57.46

D10, D50, D90: diameters at 10%, 50%, 90% cumulative volume. D [4, 3]: volume-weighted mean diameter. D [3, 2]: surface-weighted mean diameter. Span = (D90 − D10)/D50.
