# Supplementary material for: Hierarchical PLGA/PEG Barrier Engineering of Alginate Hydrogels: Scale-Dependent Burst-Release Control in Beads and Microgels
Source: Biomimetics (Basel). 2026 May 20;11(5):353. doi: 10.3390/biomimetics11050353 (PMC13204844; doi:10.3390/biomimetics11050353)
Supplement: Supplementary file 1 [file biomimetics-11-00353-s001.zip › biomimetics-4316352-supplementary.pdf]

# Supplementary Materials

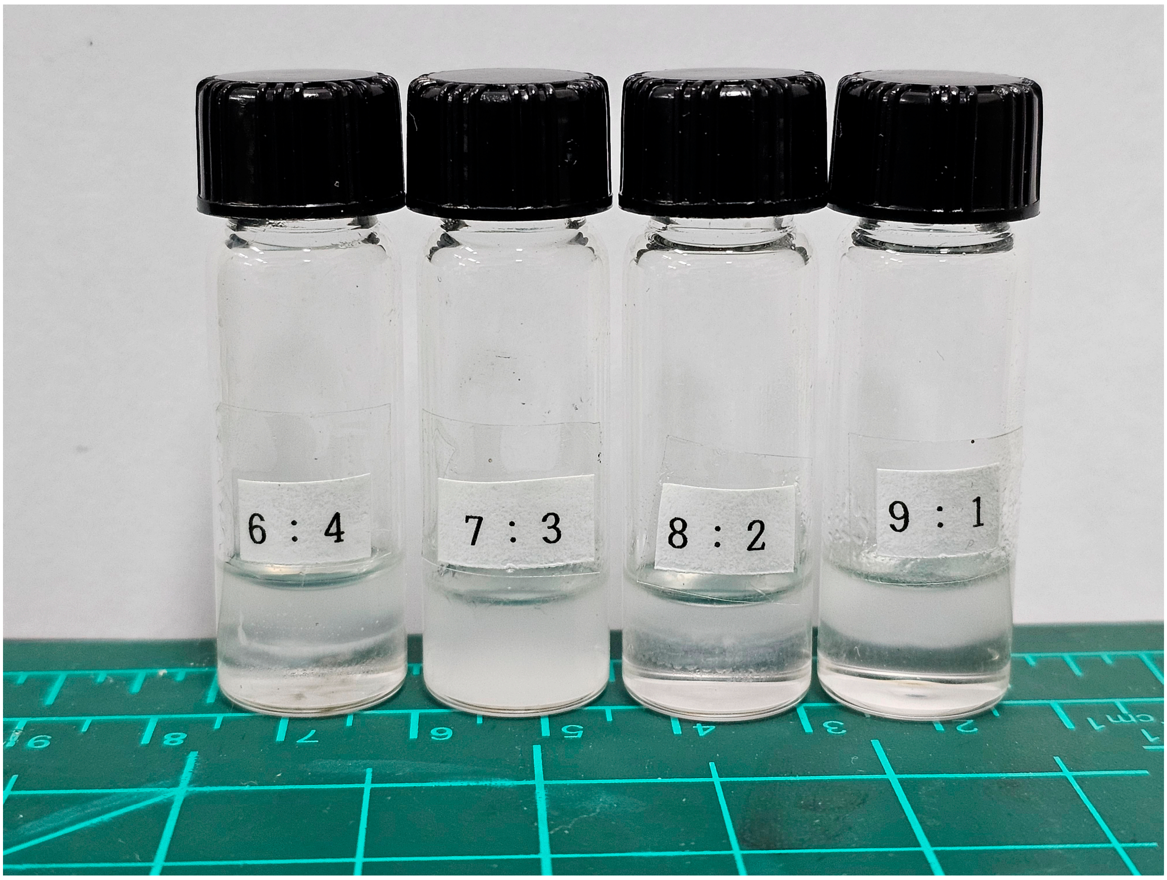


**Figure S1.** Visual comparison of macroscopic phase behavior in PLGA:PEG blend solutions at different mixing ratios (6:4, 7:3, 8:2, and 9:1 w/w) in DCM:acetone (3:1 v/v) mixed solvent at a total polymer concentration of 5 wt%. The 6:4, 8:2, and 9:1 blends all exhibited turbidity indicative of phase separation, whereas only the 7:3 blend maintained acceptable phase homogeneity. The 7:3 ratio was therefore selected as the optimal composition for dip-coating.

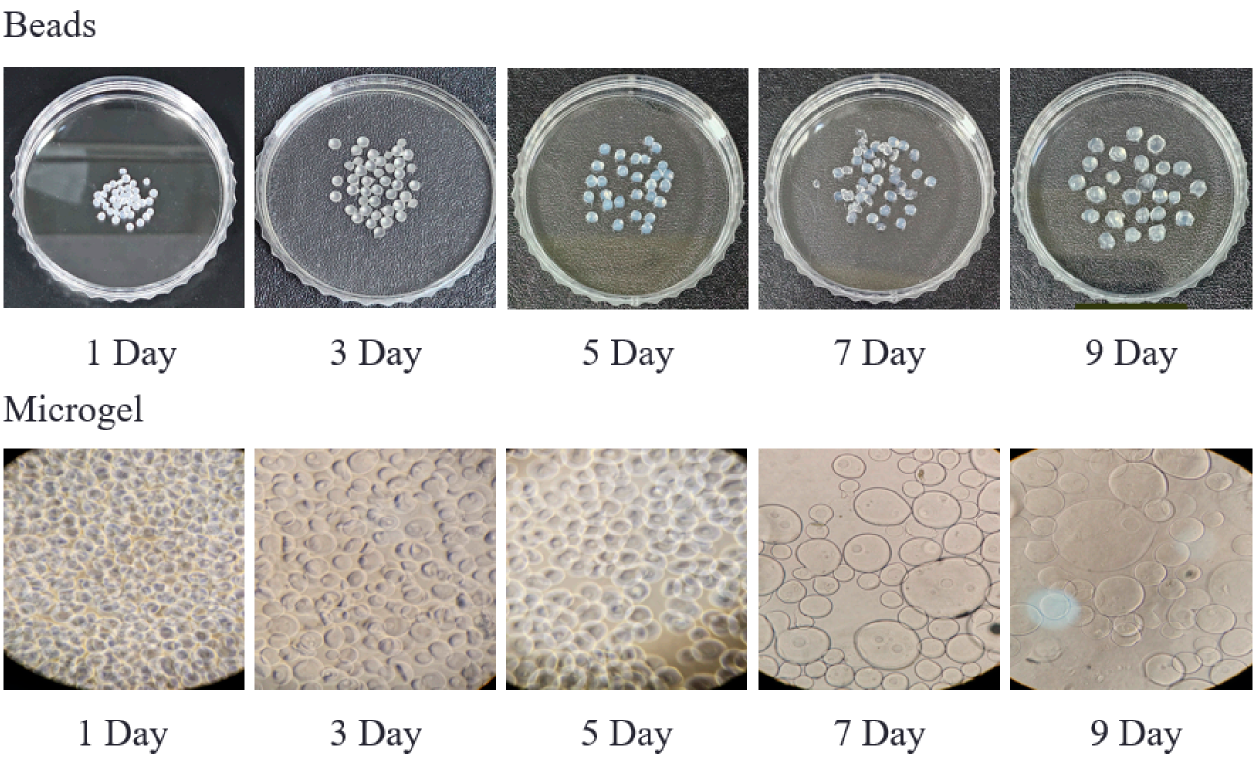


**Figure S2.** Photographs of PLGA/PEG-coated alginate beads (upper) and microgels (lower) after 1, 3, 5, 7, and 9 days of storage in PBS at 37 °C. Progressive swelling and structural degradation are visible over time; beyond 9 days, gel disintegration precluded further TGA sampling.
